# ClpA affects the virulence of *Paracidovorax citrulli* on melon by regulating RepA

**DOI:** 10.3389/fmicb.2024.1431029

**Published:** 2024-07-23

**Authors:** Shang Ziye, Zhao Yuqiang, Wu Shitong, Cai Ling, Sun Chenchao, Wang Jun, Gong Weirong, Tian Yanli, Hu Baishi

**Affiliations:** ^1^College of Plant Protection and Key Laboratory of Integrated Management of Crop Diseases and Pests, Nanjing Agricultural University, Nanjing, China; ^2^Key Laboratory of Plant Quarantine Pests Monitoring and Control, Ministry of Agriculture and Rural Affairs, Nanjing, China; ^3^Institute of Botany, Jiangsu Province and Chinese Academy of Sciences (Nanjing Botanical Garden Mem. Sun Yat-sen), Nanjing, China; ^4^Plant Protection and Quarantine Station of Province, Urumqi, China; ^5^Plant Protection and Quarantine Station of Province, Nanjing, China; ^6^Key Laboratory of Integrated Pest Management on Crops in Northwestern Oasis, Ministry of Agriculture and Rural Affairs, Xinjiang Key Laboratory of Agricultural Bio-safety, Institute of Plant Protection, Xinjiang Academy of Agricultural Sciences, Urumqi, Xinjiang, China

**Keywords:** *Paracidovorax citrulli*, ClpA, RepA, virulence, regulate

## Abstract

ClpA is a widely conserved protease in bacteria that plays a key role in virulence. To investigate its specific mechanism of action in the pathogenicity of *Paracidovorax citrulli* (formerly *Acidovorax citrulli)*, we constructed a *ClpA* deletion mutant, Δ*ClpA*. The Δ*ClpA* mutant of *P. citrulli* displayed reduced virulence on melon seedlings, and reduced motility, swarming ability, and antioxidant capacity. On the other hand, the *ClpA* deletion of *P. citrulli* mutant reduced the resistance to elevated temperature and enhanced biofilm formation ability. Using qRT-PCR, we observed that *ClpA* negatively regulates the expression of the virulence-related genes *virB*, *pilR*, *pilA*, and *fliM*, while positively regulating *hrpG*, *hrcQ*, and *trbC*. Bacterial double hybrid and Glutathione-S-transferase pulldown (GST-pulldown) results showed that ClpA interacts directly with RepA, and negatively regulates the expression of *RepA*. After deletion of the *RepA* gene, the pathogenicity of *P. citrulli* was lost, biofilm formation ability was enhanced, and the expression of *hrpG*, *pilR*, and *trbC* was positively regulated. These results indicate that ClpA plays a key role in the regulation of several virulence traits of *P. citrulli*, paving the way for future studies to better elucidate the virulence mechanisms of this bacterial plant pathogen.

## Introduction

Bacterial fruit blotch (BFB) is a plant disease that occurs worldwide (Kan et al., 2023) that can be transmitted by infecting the host or non-host plants, such as solanaceous plants (Chalupowicz et al., 2020), and has a devastating impact on species in the Cucurbitaceae family (Schaad et al., 2003; Burdman and Walcott, 2012). *Paracidovorax citrulli* (formerly *Acidovorax citrulli)*, the causal agent of BFB (Schaad et al., 1978, 2009; Willems et al., 1992; Du et al., 2023), can be spread via contaminated seeds (Hopkins and Thompson, 2002). Therefore, most BFB control measures focus on seed treatment to prevent the seed transmission of BFB. However, this approach alone cannot completely prevent BFB disease epidemics (Dutta et al., 2012). Studying the virulence mechanisms of *P. citrulli* can help us understand how the bacterium infects Cucurbitaceae host plants, provide a basis for management of BFB outbreaks, and facilitate the selection of specific pesticides or host resistance strategies.

*Paracidovorax citrulli* has multiple mechanisms for infecting plants, such as the type II secretion system (Johnson et al., 2007), the type III secretion system (Johnson et al., 2011; Ma et al., 2019; Jiménez-Guerrero et al., 2020; Ji et al., 2022), the type VI secretion system (T6SS; Tian et al., 2015), type IV pili (T4P; Bahar et al., 2009; Wang et al., 2022), flagella (Bahar et al., 2011), and quorum sensing (QS; Wang et al., 2016). Plants also have various defense mechanisms against infection, such as inducing the accumulation of reactive oxygen species to resist pathogen invasion (Zipfel, 2008; Yu et al., 2016).

As a regulatory member of the Hsp100/Clp molecular chaperone family, the ClpA protease, can bind with the ClpP protein, which has ATPase activity and acts as a catalytic subunit, to form a complex for the degradation of substrate proteins. Although ClpA and ClpP only exhibit their functions effectively when they form a complete Clp complex, these two subunits can function independently (Li and Lucius, 2013). ClpA, as an important regulatory subunit within the Hsp100/Clp molecular chaperone family, has been mainly studied in *Escherichia coli*. Its function is to recognize substrate proteins by utilizing energy from ATP hydrolysis to degrade the ClpP catalytic subunit (Zhang, 2018). ClpA is a key chaperone protein within the AAA ATPase superfamily and is involved in pathogenesis and normal growth processes of bacteria, although ClpA has been shown to be an important molecular chaperone that binds with ClpP in *Escherichia coli* (Pak and Wickner, 1997), In *Xanthomonas campestris* pv. *malvacearum* (Smith) Dye, it was found that ClpA can affect various biological phenotypes of pathogens, such as motility and colonization ability (Guo, 2018), but the functions of *ClpA* including how it affects the pathogenicity, drug resistance, and other biological traits of *P. citrulli* have not been reported.

In order to clarify the influence of *ClpA* on the pathogenesis of *P. citrulli*, *ClpA* deletion mutant was constructed, and its pathogenicity, locomotion ability, adhesion ability and HR were identified. The interaction between *ClpA* and *RepA* and other proteins was further studied, and the influence of *RepA* on *P. citrulli* was clarified, providing a new way of thinking for the prevention and treatment of BFB.

## Materials and methods

### Bacterial strains and growth conditions

Wild-type (xjL12) and mutant strains of *P. citrulli* were cultured in Luria-Bertani (LB) broth at 28°C with shaking at 220 rpm. *Escherichia coli* was cultured in LB medium, at 37°C with shaking at 220 rpm. Antibiotics were added as needed at the following concentrations: 100 ug/ml rifamycin (Rif), 50 ug/ml kanamycin (Km) and 100 ug/ml gentamicin (Gm). All bacterial strains were stored at −80°C. The specific strains and vectors used in this study are shown in Table 1.

**TABLE 1:** Bacteria and plasmids used in this study.

Bacteria strains	Relevant characteristics	Source
***Paracidovoraxcitrulli***		
xjl12	Wild-type, Rif^R^	This lab
Δ*ClpA*	*ClpA* mutant strain, containing truncated *ClpA* gene and Km cassette, Rif^R^, Km^R^	This study
Δ*ClpA*(p*ClpA*)	*ClpA* complementation strain, containing pBBR-*ClpA*, RifR, Km^R^,Gm^R^	This study
Δ*RepA*	*RepA* mutant strain, containing truncated *RepA* gene and Km cassette, Rif^R^, Km^R^	This study
***Escherichiacoli***		
DH5α	*Φ80 lacZΔM15,Δ(lacZYA-argF) U169.recA1, endA1.thi-1*	TaKaRa, Dalian, China
BW20676	Δ*pir pro hsdR, recA*	This lab
XL1-Blue MRF’Kan	D(mcrA)183, D(mcrCB-hsdSMR-mrr)173, endA1, supE44, thi-1, recA1gyrA96, relA1, lac, ^[F’proAB lacIqZDM15 Tn5 (KmR)]^	This lab
Rosetta(DE3)	F- ompT *hsd*S_B_(r_B_^–^, m_B_^–^) *gal dcm* (DE3) pRARE(*arg*U, *arg*W, *ilex, gly*T, *leu*W, *pro*L) (Cam^R^)	Beyotime, Shanghai, China
**Plasmids**		
pEX18GM	Suicide vector with a sacB gene, Gm^R^	This lab
pEX18*ClpA*Km	pEX18GM with two *ClpA* flanking fragments, Km^R^, Gm^R^	This study
pBBR1-MCS-5	Broad host range vector, Gm^R^	This lab
pBBR-*ClpA*	Containing fragment of *ClpA* and its promoter region, Gm^R^	This study
pGEX-6p-1	Plasmid used for the GST-pulldown inducing expression of proteins, Amp^R^	This lab
pET30a	Containing kanamycin cassette, Km^R^	This lab
pTRG	Plasmid used for protein expression in bacterial one-hybrid assay, Tet^R^	This lab
pBT	Plasmid used for DNA cloning in bacterial two-hybrid assay, Chlo^R^	This lab
pTRG-RepA	pTRG with the coding region of RepA, TetR	This study
pTRG-GrpE	pTRG with the coding region of GrpE, Tet^R^	This study
pTRG-SsrA	pTRG with the coding region of SsrA, Tet^R^	This study
pTRG-ClpP	pTRG with the coding region of ClpP, Tet^R^	This study
pTRG-ClpX	pTRG with the coding region of ClpX, Tet^R^	This study
pTRG-DnaJ	pTRG with the coding region of DnaJ, Tet^R^	This study
pBT-*ClpA*	pBT with the coding region of *ClpA*, Chl^R^	This study
pET30a-RepA	pET30a with the coding region of RepA, Km^R^	This study
pET30a-ClpX	pET30a with the coding region of ClpX, Km^R^	This study
pGEX-ClpA	pGEX-6p-1 with the coding region of ClpA, Gm^R^	This study

Rif^R^, Gm^R^, Km^R^, Tet^R^, Amp^R^ and Chl^R^ indicate resistance to Rifamycin, Gentamicin, Kanamycin, Tetracycline, Ampicilin and Chloromycetin, respectively.

### Construction and complementation of deletion mutants of *P. citrulli*

Using the AAC00-1 genome sequence(CP000512.1), specific oligonucleotide primers were designed to amplify the target segments (upstream and downstream fragments and Km fragment) through PCR. The PCR amplicons were assembled into pEX18GM in the order of upstream, Km, downstream to produce the recombinant vector. The recombinant vector was transformed into *P. citrulli* strain xjL12, and colonies were screened on LB agar plates containing 10% (wt/vol) sucrose, Rif (100 mg/ml), and Km (50 mg/ml) to obtain mutant colonies: Δ*ClpA* and Δ *RepA*. PCR verification was performed using primers ClpA-F1 and ClpA-R2 to select the mutants used.

Promoter prediction for the complementing genes of Δ*ClpA* was conducted using the promoter prediction website^1^, *ClpA* and its promoter were included in the design, specific primers were designed for that region, and the fragment was amplified by PCR and ligated into the pBBR1-MCS-5 vector (Kovach et al., 1995) to produce the recombinant vector. The recombinant vector was then transformed into the gene deletion mutants. Colonies were screened on LB agar plates containing Gm (50 ug/ml) and Km (50 ug/ml) to obtain the *ClpA* complemented strain. PCR verification was performed using primers ClpA-HB-F and ClpA-HB-R to select the complemented strains (Δ*ClpA*(pClpA)). The primer sequences used in this study are shown in Supplementary Table 1.

### Effect of *ClpA* on *P. citrulli* population growth dynamics

To investigate the effects of the *ClpA* mutation on bacterial population growth dynamics (Kovach et al., 1995; Wang et al., 2022). wild-type *P. citrulli* strains, *ClpA* deletion mutants, and the *ClpA* complemented strain were cultured in LB medium at 28°C, with shaking at 220 rpm overnight. The cultured strains were diluted into 25 mL of fresh LB medium to achieve a cell concentration of optical density (OD)_600_ = 0.01 and incubated at 28°C, with shaking at 220 rpm. The OD_600_ of the bacterial culture was measured every 2 h, with the measurements continuing for 36 h. This experiment was replicated three times for each bacterial strain, with each experiment was conducted three times.

### Effect of *ClpA* on *P. citrulli* virulence on melon

We use three methods to assess the role of *ClpA* in *P. citrulli* virulence, including measuring BFB symptoms on melon (cv. Huanghou) seedlings and inoculation by injection, seed-to-seedling transmission assays, and spray-inoculation on euphylla (Tian et al., 2015).

To investigate the effect of ClpA on virulence using the seedling injection assay, overnight shaking cultures of WT, Δ*ClpA*, and Δ*ClpA*(pClpA) were centrifuged to collect bacterial cells. Cells were resuspended in sterilized water and the concentration of bacterial cells were adjusted to OD_600_ = 0.3. Cells were then diluted 100,000-fold and cell suspensions were injected into the cotyledons of one-week-old melon seedlings. Ten cotyledons were injected with cell suspensions of each bacterial strain, and seedlings were incubated at 75% light intensity, 28°C and 85% humidity for four days. After incubation, BFB symptoms were observed on each cotyledon. Each strain was tested three times per experiment, with the entire set of experiments conducted three times.

To assess the role of ClpA on *P. citrulli* virulence using spray-inoculation, 20 mL of a bacterial suspension of each strain at OD_600_ = 0.3 was prepared as described above and placed in a spray bottle(LISM; Nanjing). Each strain cell suspensions were sprayed evenly on both sides of the euphylla of five melon plant,. The seedlings were then placed in plastic bags and incubated at 75% light intensity, 28°C and 85% humidity for 48 h. The bags were then removed, and the plants were incubated for four more days before observing the disease condition of the euphylla with disease index (DI) as described previously (Araújo et al., 2005), with modifications. The DI was calculated based on the formula: DI = Σ(A × B) × 100/ΣB × 5 (where A: disease class (0, 1, 2, 3, 4, 5); B: the number of seedlings in the corresponding disease class). This experiment was conducted three times.

For the seed-to-seedling transmission assays, we followed the protocol described by Tian et al. (2015). More specifically, we selected 25 germinated melon seeds of uniform growth and soaked them in 5 mL of cell suspensions (∼1 × 10^6^ CFU) of each bacterial strain for 4 h. Germinated seeds were then air-dried at room temperature. Twenty treated seeds were planted in each pot and incubated at 28°C and 85% relative humidity for 7 days. Seeds were then observed for BFB seedling symptoms. Each strain was tested three times per experiment, with the entire set of experiments conducted three times.

### Hypersensitive response assays

We investigated the influence of *ClpA* on *P. citrulli*’s ability to induce a hypersensitive response (HR) on *Nicotiana tabacum*. Each strain was cultured in LB medium until the cell suspension reached anOD_600_ = 0.3. The cell suspensions were rinsed and resuspended with sterilized water. Subsequently, 100 μL of the bacterial suspension was injected into *N. tabacum* leaves, which were then incubated at 28°C and 85% humidity for 72 h. *N. tabacum* leaves were then observed for an HR. Each strain was tested three times per experiment, with the entire set of experiments conducted three times.

### Effects of *ClpA* on *P. citrulli* twitching motility, swimming motility, and biofilm formation assays and polar flagella morphology

The twitching motility assay was conducted as previously described by Bahar et al. (2009). Specifically, the concentration of bacterial suspensions was adjusted to OD_600_ = 0.3 with double-distilled water and diluted 10,000-fold. Then, the cell suspension was spread onto 1% nutrient agar (NA) medium and cultured at 28°C for 48 h. A stereo fluorescence microscope (Nikon) was used to observe twitching motility (wave-like ripples around a single colony).

The swimming motility assay was conducted as previously described by Liu et al. (2019). Bacterial cell suspensions were adjusted to OD_600_ = 0.3 using LB liquid medium, and 2 μL of the suspension was inoculated into the center of a 0.3% motility agar plate (Liu et al., 2019). After incubation at 28°C for 48 h, quantitative analysis was made by bar chart with the diameter of the swimming motility zone was measured. Each strain was tested three times per experiment, with the entire set of experiments were conducted three times.

Biofilm assays were conducted as previously described (Liu et al., 2019). Liquid LB medium was used to adjust bacterial cell suspensions to OD_600_ = 1.0. Then, 4 mL of LB liquid medium was added to each well of a 12-well PVC plate (Polyvinyl chloride plate; BeyoGold™ 12 Well Cell Culture Plates; Shanghai), and 40 μL of bacterial suspension was inoculated into it. The plate was incubated at 28°C for 48 h and after decanting the supernatant, the OD_600_ was measured. The PVC plate was then dried in an 80°C oven for 20 min. Five milliliters of 1% crystal violet was added to each well, incubated for 45 minutes, and then the purple precipitate in the well was photographed. The biofilm was dissolved with 5 mL of anhydrous ethanol and the OD_590_ was measured. The OD_590_/OD_600_ ratio was calculated. We used transmission electron microscopy (TEM) to visualize polar flagella of *P. citrulli*, as described by Bahar et al. (2009). Each strain was tested three times per experiment, with the entire set of experiments were conducted three times.

### Effect of *ClpA* on *P. citrulli* H_2_O_2_ sensitivity and heat resistance

The sensitivity of *P. citrulli* to hydrogen peroxide (H_2_O_2_) was determined by measuring the diameter of the inhibition zones on agar plates with different concentrations of H_2_O_2_. Bacterial strains were incubated at 28°C, 220 rpm overnight to reach an OD_600_ = 0.3. Then, 2 mL of the adjusted bacterial culture was transferred to 98 mL of molten LB medium at 40°C, poured into Petri dishes and allowed to solidify. A sterilized filter paper disc (5 mm diameter) was placed in the center of each agar plate, and 5 μL of different concentrations (1%, 5%, 10%) of H_2_O_2_ were added to the filter paper. Filter discs were incubated at 28°C for 48 h and then the diameters of the inhibition zones were measured.

Bacterial strains were incubated at 28°C, 220 rpm overnight to reach an OD_600_ = 0.3 then 1 mL of the each adjusted bacterial culture was transferred to different test tubes. The test tubes were then incubated in water bath at different temperatures (35°C, 40°C, 45°C, 50°C, 55°C, 60°C), for 10 min, and 0.1 ml of bacterial suspension was re-injected into 5 ml LB medium, and shaken at 28°C for 24 h. Bacterial population growth was monitored by measuring OD_600_. Quantitative analysis was made by bar chart with the OD_600_ was measured. This experiment was conducted three times for each bacterial strain and the study was conducted three times.

### RNA isolation and quantitative real-time PCR analysis

Bacterial strains were cultured at 28°C, at 220 rpm to a concentration of OD_600_ = 1.0, then bacterial cells were harvested by centrifugation at 1000 × g for 1 min. Total bacterial RNA was extracted using a bacterial RNA extraction kit (Pudi DP201; Shanghai). The extracted RNA was reverse transcribed using HiScript III RT SuperMix with gDNA Wiper (Vazyme; Nanjing) to obtain cDNA. The cDNA was diluted to 50ng/μL and real-time fluorescent quantitative PCR was conducted on a ABI PRISM 7500 real-time PCR machine (Applied Biosystem) using ChamQ Universal SYBR qPCR Master Mix (Vazyme; Nanjing). The reference gene was the 16S ribosomal RNA of *P. citrulli*. The qRT-PCR program was as follows: 95°C for 30s, 95°C for 10s, 60°C for 30s, for 40 cycles, followed by a final melting curve analysis from 60 to 95°C. Each gene had three biological replicates, and the above experiments were conducted three times. The final gene expression level changes were based on the 2^–ΔΔCt^ value.

### Bacterial two-hybrid experiments

Bacterial two-hybrid assays were used to investigate the interaction between ClpA and putative targets (Table 1). The coding region (2,352 bp) of ClpA was linked to pTRG to form the recombinant vector pTRG-ClpA. The coding regions of the putative target proteins were linked to pBT to form recombinant vectors pBT-ClpP, RepA, etc. The pTRG and pBT recombinant vectors were co-transformed into the XL1-Blue MRF′ Kan strain. When ClpA interacts with the putative target, the transformed *E. coli* strain will grow on the selection medium (minimum medium with 5 mm 3-amino-1,2,4-triazole, streptomycin 8 ug/ml, tetracycline 12.5 ug/ml, chloramphenicol 34 ug/ml, and Km 30 ug/ml) (Wang et al., 2018). Colony growth was observed on the medium after 48 h of incubation at 28°C. pBT and pTRG were used to co-transform strains as positive control and pBT-target with pTRG empty vector strains as negative control.

### Gst-pull down assay

pET330a-RepA-His and pGEX-4T-1-ClpA protein expression vectors were constructed and transformed into *E. coli.* Rosetta(DE3). Cells were inoculated into LB medium containing 50 μg/mL Amp and incubated overnight at 37°C with shaking. 100 μL of the culture was transferred into 25 mL of LB medium containing 50 μg/mL Amp and shaken at 37°C until the OD_600_ = 0.6. IPTG was added to the culture to a final concentration of 0.5 mM, and the culture was shaken at 16°C for over 7 hours. The cells were harvested and resuspend in PBS, lysed by sonication, and centrifuged to collect the supernatant.

GST-ClpA and His-RepA proteins were mixed in a 1:1 ratio, and 50 μL of the mixture add 12.5 μL of 5 × loading buffer were added. The mixture was then boiled at 98°C for 10 min and used as the input sample. 20 μL of glutathione Sepharose 4B was added to the remaining mixture, which was then rotated and mixed at 4°C for 4 h, centrifuged at 500 × g for 5 min. to remove the supernatant, and the pellet was resuspended in 1mL of high salt PBST (250 mL phosphate-buffered saline + 1% Tween + 3 g NaCl), and mixed by rotating at 4°C for 10 min. The mixture was centrifuged at 500 × g for 5 min, the supernatant was removed, and the washing process was repeated three times. 50 μL glutathione reductase was added to the final pellet, and after mixing, the sample was centrifuged at 4°C, 500 × g for 5 min. 50 μL of the supernatant was mixed with 12.5 μL of 5 × loading buffer, boiled at 98°C for 10 min. and used as the output sample. A GST empty vector was used as the control.

The final samples were analyzed by Sodium Dodecyl Sulfate-Polyacrylamide Gel Electrophoresis (SDS-PAGE) and transferred onto polyvinylidene fluoride membranes (Millipore, Red Bank, NJ, USA) using a semi-dry protein transfer system. Membranes were blocked with 5% milk at room temperature for 1 h, washed with 5% Tween Tris-buffered saline (TBST) solution at pH 7.5, and incubated with specific anti-GST antibodies and His tags for 1 h, followed by incubation with anti-rabbit secondary antibodies for 1 h. Protein immunoblotting was performed using the HyGlo HRP ECL detection kit (MDBio Inc., Qingdao, China) and membranes were photographed using the Tanon-6600 automatic multifunctional imaging analysis system (Tanon, Shanghai, China).

## Results

### *ClpA* does not affect bacterial growth, but it attenuates the virulence of *P. citrulli*

The ClpA protein of *P. citrulli* is closely related to the protein in bacteria in the *P. citrulli* (WP017438384.1:1-783) (Supplementary Figure 1). However, the absence of ClpA did not affect in vitro growth of *P. citrulli* (Supplementary Figure 2A). To determine the impact of *ClpA* on the virulence of *P. citrulli*, we compared the severity of BFB symptoms on melon cotyledons inoculated with WT, Δ*ClpA*, and the complementary strain, Δ*ClpA*(pClpA).

**FIGURE 1:**
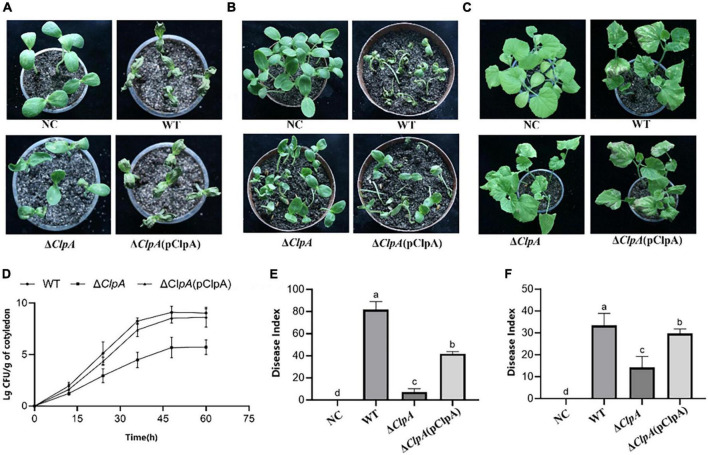
Role of *ClpA* in *Paracidovorax citrulli* virulence and bacterial growth in melon seedlings. **(A)** Melon seedling cotyledons inoculated by injection with *P. citrulli* wild-type (WT): xjL12, Δ*ClpA*, and Δ*ClpA* (pClpA) strains (∼1 × 10^3^ CFU/ml), and double-distilled H_2_O as a negative control (NC). Seedlings were observed for bacterial fruit blotch symptoms at 4 days post-inoculation (dpi). **(B)** Melon seeds were inoculated by soaking in bacterial cell suspensions (∼1 × 10^6^ CFU/ml) of *P. citrulli* WT, Δ*ClpA*, and Δ*ClpA* (pClpA). BFB symptoms were observed 7 days after planting. **(C)** Spray-inoculation of melon euphylla with *P. citrulli* cell suspensions (∼1 × 10^6^ CFU/ml) of WT, Δ*ClpA*, and Δ*ClpA* (pClpA). **(D)** Bacterial suspensions (∼1 × 10^3^ CFU/ml) of each strain were injected into cotyledons of melon seedlings and bacterial populations were quantified at 0, 12, 24, 48, and 60 h post-inoculation. **(E)** Melon seed were inoculated with *P. citrulli* strains (∼1 × 10^3^ CFU/ml) planted under conditions conducive for BFB development and the disease index of seed-seedling transmission were determined. The letters above the bars represent significant differences (*P* < 0.05, LSD). **(F)** 7 days after spray-inoculation on euphylla and the disease index of spraying inoculation on euphylla was determined. The letters above the bars represent significant differences (*P* < 0.05, LSD).

Compared to the WT, the cotyledons of melon seedlings inoculated with the Δ*ClpA* strain did not induce BFB symptoms (Figure 1A), and *P. citrulli* virulence was restored in the complemented strain. We also found that the average bacterial cell populations of *P. citrulli* WT, Δ*ClpA*, and Δ*ClpA* (pClpA) strains were approximately 1.26 × 10^9^, 4.78 × 10^5^, and 3.34 × 10^8^ CFU/g of tissue, respectively, by 48 hpi (Figure 1D). In the other two virulence assays, the *ClpA* mutants of *P. citrulli* also displayed reductions of BFB symptoms (Figures 1B,C). In the seed-to-seedling transmission assays, the disease index of the negative control (NC) was 0, while that of WT, Δ*ClpA*, and the complemented strain were 81.6, 7.3, and 41.9, respectively (Figure 1E). In the true leaf spray-inoculation experiment, the disease indices for NC, WT, Δ*ClpA*, and the complemented strain were 0, 33.4, 14.3, and 29.8, respectively (Figure 1F). Compared to WT, the virulence of Δ*ClpA* on the cotyledons of melon seedlings was significantly reduced. The virulence of *P. citrulli* Δ*ClpA* on true leaves was also significantly decreased, as was its seed-to-seedling-transmission ability (*P* < 0.05).

### *ClpA* attenuates *P. citrulli* swimming motility, twitching motility and enhances biofilm production, and positively regulates expression of genes related to flagella and pili

**FIGURE 2:**
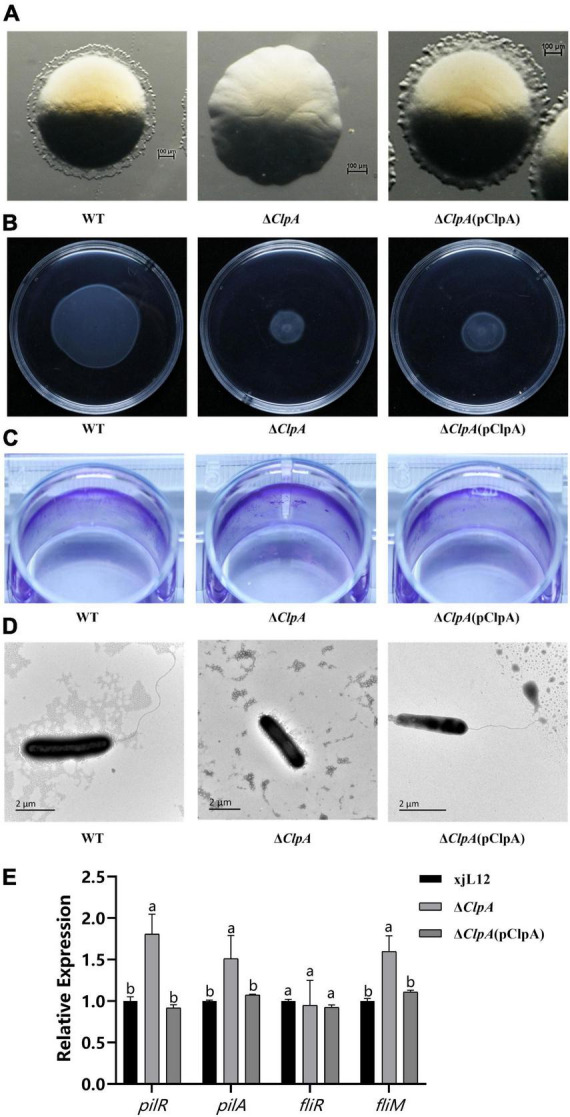
Role of *ClpA* in twitching motility, swimming motility, and biofilm formation of *P. citrulli*. **(A)** Twitching motility of *P. citrulli* strains including wild-type (WT): xjL12, Δ*ClpA*, and Δ*ClpA* (pClpA). Strains were transferred onto 1% NA medium and the resulting colonies were photographed using a stereoscope after 48 h. **(B)** Swimming motility of *P. citrulli* strains including WT: xjL12, Δ*ClpA*, and Δ*ClpA* (pClpA) on 0.3% agar plates at 28°C for 2 days. **(C)** Biofilm formation of *P. citrulli* strains including WT: xjL12, Δ*ClpA*, and Δ*ClpA* (pClpA). Biofilm production is indicated by a ring of dark purple precipitate on the inner wall of culture plate wells. **(D)** Transmission electron microscope verification of presence polar flagella. Full-length flagella (arrows) can be seen in WT and Δ*ClpA* (pClpA) strains, while flagella were not visible for Δ*ClpA*. **(E)** Expression level of pili and flagella-related genes *pilR, pilA, fliR* and *fliM* in *P. citrulli* WT, Δ*ClpA* and Δ*ClpA* (pClpA) were determined by qRT-PCR. Different lowercase letters indicate a significant difference between treatments. Statistically significant differences were determined by the one-way ANOVA of variance and *P* < 0.05.

In the twitching motility assay, after 48 h of cultivation at 28°C, wave-like halos surrounding single colonies were visible under the stereo microscope on NA plates, however, the swarming ring of Δ*ClpA* was less than that of the WT strain (Figure 2A). The swimming motility phenotype of Δ*ClpA* was less than that of WT (Supplementary Figure 5A; *P* < 0.05), and the complemented strain did not restore the function (Figure 2B). The biofilm formation ability of Δ*ClpA* was enhanced relative to the WT strain (Figure 2C; Supplementary Figure 4A), and using transmission electron microscopy, we observed full-length polar flagella for WT and Δ*ClpA* (pClpA) strains, but not for Δ*ClpA* (Figure 2D).

Based on qRT-PCR results, we found that the expression levels of *pilR* and *pilA*, two genes related to pili, can affect the pathogenicity of *P. citrulli* (Yang et al., 2023), were significantly upregulated. This is consistent with the enhanced biofilm formation observed in the biofilm assays. However, the expression levels of the gene associated with flagellum of *P. citrulli* (Zhang, 2018), *fliM*, were significantly upregulated, and there was no difference in expression of *fliR* (Figure 2E).

### *ClpA* deletion weakened the antioxidative capacity and reduced the resistance to elevated temperature of *P. citrulli*

**FIGURE 3:**
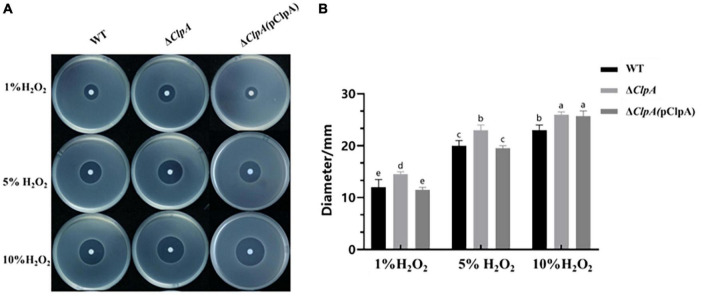
Sensitivity of WT, Δ*ClpA* and Δ*ClpA*(pClpA) strains to H2O2. **(A)** 2 μL of different concentrations (1, 5, and 10%) of H_2_O_2_ were dropped in the center of the plates. The H_2_O_2_inhibition zones were observed and measured after incubation at 28°C for 48 h. **(B)** The diameter of the zone of bacterial growth inhibition. Experiments were performed in triplicate and were repeated three times with similar results. Data represent the means of three replicates ± standard deviations (error bars). Different lowercase letters indicate a significant difference between treatments. Statistically significant differences were determined by the one-way ANOVA and *P* < 0.05.

To elucidate the role of *ClpA* in the antioxidative capacity of *P. citrulli*, the inhibition zone method was used to measure sensitivity of WT, Δ*ClpA*, and complemented strains to different concentrations of hydrogen peroxide. Compared to WT, the diameter of the inhibition zone of Δ*ClpA* was significantly larger (Figure 3B; *P* < 0.05), indicating increased sensitivity to H_2_O_2_ (Figure 3A).

In the high-temperature tolerance experiment, we found that at 35°C, there was no significant difference in the number of surviving bacteria cells between the WT and Δ*ClpA* mutant. However, at 40°C, the WT had significantly more surviving bacterial cells than the Δ*ClpA* mutant. The WT strain and Δ*ClpA* (pClpA) strain survived at 45°C and the number of bacterial cells increased, while the Δ*ClpA* mutant did not survive. After incubating at 50°C, 55°C, and 60°C for 10 min, WT, Δ*Clp* and Δ*ClpA* (pClpA) was unable to grow. This indicates that *ClpA* affects *P. citrulli*’s sensitivity to high temperatures (Supplementary Figure 3), *ClpA* deletion reduced the resistance to elevated temperature of *P. citrulli*.

### *ClpA* does not influence the ability of *P. citrulli* to induce HR on *N. tabacum*, but positively regulates expression of genes related to pathogenicity

**FIGURE 4:**
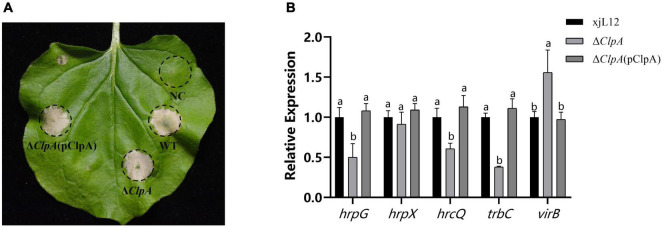
Effects of *ClpA* on *Nicotiana tabacum* hypersensitive response (HR) induction by *P. citrulli* strains. **(A)** Tobacco HR induction. Approximately 100 μl of bacterial suspension (OD_600_ = 0.3) was infiltrated at each inoculation site on a tobacco leaf and the plant was incubated at 28°C. *N. tabacum* was grown under greenhouse conditions at 24°C and observed for HR at 24 to 72 h post-infiltration(hpi). Negative Control: double-distilled H_2_O; WT: wild-type xjl12; Δ*ClpA*; Δ*ClpA*(pClpA). **(B)** Expression level of *hrpG, hrpX, hrcQ, trbC and virB* genes in WT, Δ*ClpA* and Δ*ClpA*(pClpA) strains by qRT-PCR. Experiments were conducted in triplicate and repeated three times with similar results. Data shown represent the means of three replicates ± standard deviations. Error bars indicate standard deviations. Different lowercase letters indicate a significant difference between strains. Statistically significant differences were determined by the one-way Analysis of variance (ANOVA) and *P* < 0.05.

This study investigated the expression levels of related pathogenic genes in Δ*ClpA* to clarify how *ClpA* impacts pathogenicity of *P. citrulli*, *hrcQ, hrpG* and *hrpX* has been reported in *P. citrulli*, which is an important T3SS regulatory gene (Bahar and Burdman, 2010; Burdman and Walcott, 2012; Yu, 2018). *trbC* and *virB* is the gene that affects the assembly of pili in T4SS (Christie et al., 2017; Shala-Lawrence et al., 2018). The results showed that after the deletion of *ClpA*, we found that the expression levels of *hrpG* (T3SS gene), *hrcQ* (T3SS gene), and *trbC* (T4SS), were significantly downregulated, while *virB*(T4SS gene) was upregulated (Figure 4B). Δ*ClpA* and Δ*ClpA*(pClpA) did not influence the ability of *P. citrulli* to induce a HR on *Nicotiana tabacum* (Figure 4A).

### ClpA interacts with RepA but not with ClpP

**FIGURE 5:**
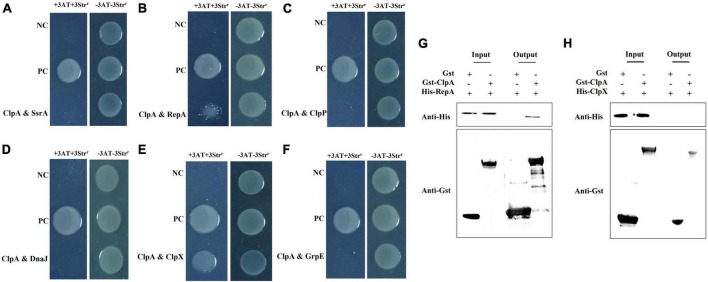
Bacterial two-hybrid assays: pTRG-GacSand pBT-GacS: co transformant containing pTRG-GacS and pBT-GacS serves as a positive control; 3AT-Str^r^: no selective LB medium plate; and +3AT + Str^r^: M9-based selective medium plate. **(A)** ClpA and SsrA displayed no interaction as verified by bacterial two-hybrid assay. ssrA was cloned into vector pTRG, and ClpA were cloned into vector pBT, respectively. **(B)** ClpA and RepA interaction verified by bacterial two-hybrid. RepA was cloned into vector pTRG, and ClpA were cloned into vector pBT, respectively. **(C)** ClpA and ClpP showed no interaction as verified by bacterial two-hybrid assay. ClpP was cloned into vector pTRG, and ClpA were cloned into vector pBT, respectively. **(D)** ClpA and DnaJ no interaction verified by bacterial two-hybrid. DnaJ was cloned into vector pTRG, and ClpA were cloned into vector pBT, respectively. **(E)** ClpA and ClpX interaction verified by bacterial two-hybrid. ClpX was cloned into vector pTRG, and ClpA were cloned into vector pBT, respectively. **(F)** ClpA and GrpE no interaction verified by bacterial two-hybrid. GrpE was cloned into vector pTRG, and ClpA were cloned into vector pBT, respectively. **(G)** Interaction between *P. citrulli* ClpA and RepA is indicated by a GST pull-down assay. The purified GST-ClpA was incubated with His-RepA and pulled down with GST beads. **(H)** Interaction between *P. citrulli* ClpA and ClpX is indicated by a GST pull-down assay. The purified GST-ClpA was incubated with His-ClpX and pulled down with GST beads.

**FIGURE 6:**
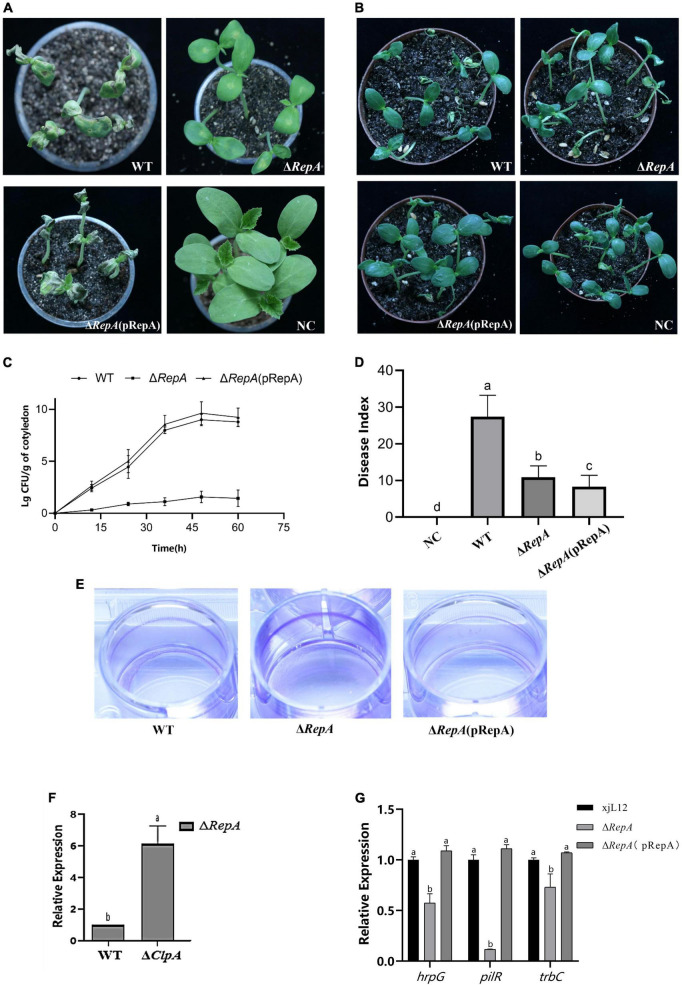
Role of *RepA* in *P. citrulli* virulence and bacterial growth in melon seedlings. **(A)** Melon seedling cotyledons inoculated by injection with *P. citrulli* wild-type (WT): xjL12, Δ*RepA* and Δ*RepA*(pRepA) strains (∼1 × 103 CFU/ml), and double-distilled H_2_O (ddH_2_O) as a negative control (NC). Seedlings were observed for bacterial fruit blotch symptoms at 4 days post-inoculation (dpi). **(B)** Melon seeds were inoculated by soaking in bacterial cell suspensions (∼1 × 10^6^ CFU/ml) of *P. citrulli* WT, Δ*RepA*,Δ*RepA*(pRepA). BFB symptoms were observed 7 days after planting. **(C)** Bacterial suspensions (∼1 × 10^3^ CFU/ml) of each strain were injected into cotyledons of melon seedlings and populations were quantified at 0, 12, 24, 48, and 60 h post-inoculation. **(D)** Melon seeds were inoculated with *P. citrulli* strains (∼1 × 10^3^ CFU/ml) planted under conditions conducive for BFB development and the disease index of seed-seedling transmission were calculated. The letters above the bars represent Least Significant Differences (*P* < 0.05, LSD). **(E)** Biofilm formation of P. citrulli strains including wild-type (WT): xjL12, ΔRepA and ΔRepA(pRepA). Biofilm production is indicated by a ring of dark purple precipitate on the inner wall of culture plate wells. **(F)** Expression level of *RepA* between *P. citrulli* WT and Δ*ClpA* were determined by qRT-PCR. Different lowercase letters indicate a significant difference between treatments. Statistically significant differences were determined by the one-way ANOVA of variance and *P* < 0.05. **(G)** Expression level of pathogenicity-related genes *hrpG, pilR, trbC* between *P. citrulli* WT, Δ*RepA* and Δ*RepA*(pRepA)were determined by qRT-PCR. Different lowercase letters indicate a significant difference between treatments. Statistically significant differences were determined by the one-way analysis of variance and *P* < 0.05.

Bacterial two-hybrid assays were conducted targeting proteins closely related to ClpA and pathogenic genes to elucidate the interaction targets of ClpA and understand how it impacts *P. citrulli* pathogenicity. Based on to Pak’s study (Pak and Wickner, 1997) and our preliminary results, we selected six targets: SsrA: the polypeptide translated from truncated mRNAs that are marked by a short peptide, known as SsrA tag, at their C-termini and directed to the specific endogenous proteases for C-terminal proteolysis; RepA: A protein that recognizes the origin of replication (ori) in the plasmid; ClpX: Chaperone protein; ClpP: Catalytic subunit; DnaJ: Heat shock associated chaperone proteins; and GrpE: Essential protein for resistance to high temperature stress. Using a two-hybrid assay between ClpA and potential targets, we found that in *P. citrulli*, there is an interaction between RepA, ClpX with ClpA (Figures 5B, E), but ClpA does not interact with SsrA, DnaJ, GrpE (Figures 5A, D, F). Although ClpA exerts its full function by forming a complex with ClpP in many bacteria, in *P. citrulli*, ClpA does not interact with ClpP (Figure 5C). Therefore, we assumed that ClpA directly interacts with RepA. To further validate the regulatory relationship between ClpA and its interaction targets, Gst pull-down experiments were performed. (Figure 5G), this result showed that ClpA directly interacts with RepA, but ClpA does not directly interact with ClpX(Figure 5H). qRT-PCR results indicated that in Δ*ClpA*, the expression levels of *RepA* were significantly upregulated, meaning that *ClpA* negatively regulates *RepA*(Figure 6F).

### *RepA* attenuates the pathogenicity of *P. citrulli*, enhances biofilm production and reduces the expression of pathogenicity-related genes.

Preliminary verification of the effect of *RepA* on the pathogenicity of *P. citrulli* was obtained through virulence assays on melon seedlings. In both pathogenicity assays, the mutant did not cause BFB symptoms. Compared to the WT, the cotyledons of melon seedlings inoculated with the Δ*RepA* strain showed significantly reduced BFB symptoms, and the complementary strain Δ*RepA*(pRepA) displayed symptoms similar to the WT strain (Figure 6A). The average bacterial cell populations for *P. citrulli* WT, Δ*RepA*(pRepA) and Δ*Rep* strains were approximately 1 × 10^9^, 4 × 10^9^ and 37 CFU/g of tissue, respectively, by 48 hpi (Figure 6C). In the seedling transmission test, the disease indexes for seeds inoculated with NC, WT, Δ*RepA*(pRepA) and Δ*RepA* were 0, 27.4, 8.3 and 10.9 (Figure 6D), respectively, after 7 days of cultivation. Compared to WT, Δ*RepA* showed significantly reduced virulence on melon cotyledons and a significant reduction in seedling transmission (*P* < 0.05; Figure 6B), and the complementary strain Δ*RepA*(pRepA) did not recover pathogenicity. These results suggest that *RepA* is the key to *P. citrulli* pathogenicity.

This study determined the role of *RepA* by measuring the biofilm production and swimming motility of *P. citrulli*. In the motility assay, the diameter of Δ*RepA* was not different from that of the WT strain (Supplementary Figure 5C). However, in the biofilm assay, the biofilm formation ability of Δ*RepA* significantly increased (Figure 6E), which was similar to Δ*ClpA*. These results suggest that *RepA* is the key to *P. citrulli* biofilm formation. *ClpA* can regulate the expression of *RepA* (Figure 6F). To clarify how *RepA* affects the pathogenicity of *P. citrulli*, we quantified the expression of pathogenicity-related genes in Δ*RepA* and found that the expression levels of *hrpG*, *pilR, trbC* were significantly downregulated (Figure 6G). In contrast, the expression levels of *RepA* was significantly upregulated, while *pilA, fliM, virB* were not changed (Supplementary Figure 5D). These results suggest that *RepA* is important for positively regulating the expression levels of the T3SS gene (*hrpG*), pili gene (*pilR*) and quorum gene (*trbC*) of *P. citrulli* (Figure 6G). So *RepA* is the key to the pili, T3SS and quorum sensing.

## Discussion

The AAA ATPase superfamily in *P. citrulli* has a key impact on bacterial virulence, such as the deletion of genes ClpP, which also leads to a significant reduction in pathogenicity of *P. citrulli* (Yu, 2023). As a member of the Hsp100/Clp family, ClpA is responsible for preparing protein substrates to be degraded by ClpP (Butler et al., 2006). In this study, by constructing a *ClpA* deletion mutant strain of *P. citrulli* and its corresponding complemented strain, we found that although ClpA is essential for normal bacterial life (Gottesman, 1996), its absence does not affect the growth of *P. citrulli*. However, Δ*ClpA* displayed significantly reduced pathogenicity in cotyledon-injection assays, true leaf spray-inoculation assays, and seedling transmission assays. *P. citrulli* pathogenicity involves systems such as T3SS (Johnson et al., 2011), quorum sensing (Wang et al., 2016) and flagella (Bahar et al., 2011). The melon host plant’s PTI/ETI also impedes bacterial infection by inducing ROS bursts (Dodds and Rathjen, 2010; Block and Alfano, 2011). Therefore, in this study, the in vitro motility, adhesion ability, and antioxidant capacity of various strains were studied. The results showed that Δ*ClpA* motility halo disappeared, and the complemented strain’s halo was restored. While the mutant lost swimming motility, the complemented strain did not fully recover his phenotype. These results are similar to reports on ClpX and ClpP (Yu, 2023). This study validated the changes of high-temperature resistance of Δ*ClpA* and found that bacterial resistance to high temperatures was weakened after the deletion of the *ClpA* gene. This result is in accordance with earlier study which showed that *ClpA* is essential for bacterial survival under high-temperature conditions (Lo et al., 2022). In order to identify potential interacting targets for ClpA, we chose GrpE and DnaJ, which are proteins related to bacterial response to high temperatures (Thomas and Baneyx, 2000), for bacterial two-hybrid validation. However, there are no interaction between ClpA and GrpE, DnaJ. Therefore, we assumed that ClpA could play an independent role in *P. citrulli*, and further experiments will be conducted to verify this hypothesis. In in vitro adhesion ability assays, the biofilm formation ability of Δ*ClpA* was significantly enhanced, and the biofilm formation phenotype of the complemented strain was restored. Research has indicated that bacterial biofilms play a role as virulence factors in bacterial pathogenicity (Jain and Agarwal, 2009). Li’s study was observed that there is a significant difference in bacterial pathogenicity between biofilm of *Pseudomonas aeruginosa* and planktonic forms of *Pseudomonas aeruginosa*, with gene expression related to pathogenicity being approximately 30 times lower in biofilm bacteria compared to the planktonic counterparts, this study also revealed that the formation of bacterial biofilms affects the release of virulence factors by planktonic bacteria, resulting in reduced pathogenicity. Furthermore, it was discovered that biofilms possess inherent immunity against host responses, which could contribute to this decrease in bacterial toxicity (Li et al., 2014), however, the real reason for this phenomenon in *P. citrulli* still needs to be verified by further experiments. *ClpA* is a key gene in *P. citrulli* pathogenicity and it may have complex interactions with neighboring genes. This may explain why the complemented strain could not fully restore the biofilm phenotype.

The enhanced biofilm formation ability of Δ*ClpA* in this study is opposite to the *ClpX* deletion in *P. citrulli* and *ClpP* deletion in *P. citrulli* (Yu, 2023), so we speculate that ClpA may not regulate in the traditional binding form to form a complex with ClpP in *P. citrulli*. To clarify the regulation of virulence by *ClpA* in *P. citrulli*, we performed real-time quantitative PCR assays to measure the expression of related pathogenic genes in Δ*ClpA* and found that the expression levels of *pilR, pilA, fliM, and virB* genes were significantly upregulated, while *hrpG, hrcQ*, and *trbC* were significantly downregulated. There was no significant change in the expression levels of *hrpX* and *fliR*. Similar results were observed with the deletion of *ClpA* in Aac5 (Yu, 2023); so ClpA is involved in the regulation of pathogenic genes’ expression in *P. citrulli.* To further clarify how *ClpA* affects the expression of related genes, we screened for potential interaction targets of *ClpA*. Through bacterial two-hybrid assays, we confirmed the interaction of ClpA with RepA, ClpA may has indirect interaction with ClpX(further experimental verification is required), and the absence of interaction with the catalytic subunit ClpP. This observation agrees with our previous conclusion, but in *P. citrulli*, *ClpA* can regulate the expression of ClpP (Yu, 2023). We further speculate that ClpA plays a role in protein degradation independently, and can regulate RepA to influence the degradation of ClpP and the other proteins in *P. citrulli*.

Using GST-pulldown assays we found that ClpA directly interacts with RepA as reported by Pak and Wicker (Pak and Wickner, 1997). We also observed that the expression levels of *RepA* were significantly upregulated in Δ*ClpA*. We observed that *RepA*’s expression level is significantly enhanced, but Pak and Wickner showed that ClpA can activate RepA in *E.coli* (Pak and Wickner, 1997), which may be different to *P. citrulli*. Research has shown that ClpA can remodel the RepA dimer of bacteriophage P1 into a monomer, activating the potential DNA binding activity of RepA (Wickner et al., 1994; Pak and Wickner, 1997). When independent of ClpP, ClpA functions as an ATP-dependent chaperone protein to activate the P1 plasmid replication initiation protein RepA in vitro, similar to DnaJ and GrpE (Wickner et al., 1994). Although it can highly activate specific DNA binding sites for RepA, it is not part of the RepA-specific DNA complex (Wickner et al., 1991). ClpA also protects RepA from heat-induced inactivation and prevents destructive thermal denaturation outside of luciferase bodies (Wickner et al., 1994). Therefore, we constructed a *RepA* deletion mutant to investigate the impact of *RepA* on *P. citrulli* virulence and to determine whether *ClpA* influences the pathogenicity by interacting with *RepA*. The results showed that the growth ability of Δ*RepA* did not change significantly, but its virulence, including symptoms on melon seedlings in injection assays, and seed-to-seedling transmission assays, was reduced, which is consistent with the phenotype of Δ*ClpA*. Hou et al. showed that the resistance response induced by *RepA* in *N. tabacum* is temperature-sensitive (Hou, 2015), and this was true for Δ*ClpA* and Δ*RepA*.

When testing the biofilm formation ability of Δ*RepA*, we observed an increased biofilm formation ability, which also aligns with the phenotype of Δ*ClpA*. Therefore, we conducted quantitative analyses of expression of virulence-related genes that showed significant changes in Δ*ClpA* and discovered in Δ*RepA*, genes such as *hrpG*, *pilR*, and *trbC* were significantly downregulated. This indicated that *RepA* positively regulates the expression of genes related to virulence and pili formation. We observed that the change in expression of *hrpG*, *trbC* in Δ*RepA* was similar for Δ*ClpA*. However, the change in expression of *pilR*, which is downregulated in Δ*RepA*, is upregulated in Δ*Clp*. Therefore, *ClpA* negatively regulates the expression of *pilR* and *RepA* positively regulates the expression of *pilR*. In other words, *ClpA* can regulate *RepA* to change the expression of *pilR*, then change the pili and pathogenicity. In contrast, the biofilm formation ability of Δ*RepA* increased; it may be regulated by more genes than a single *pilR*. More research is needed to better understand this phenomenon.

**FIGURE 7:**
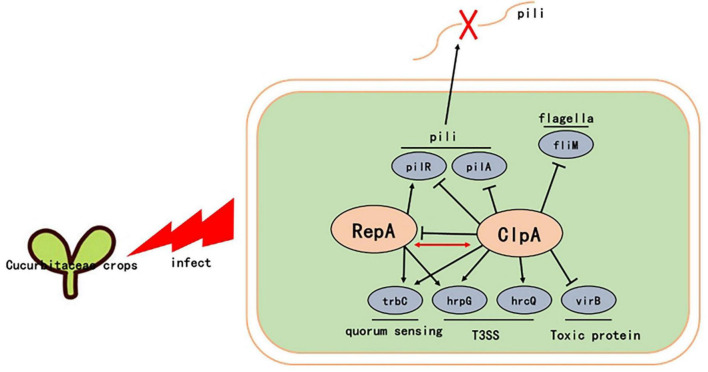
Proposed model illustrating the global effect of *ClpA* in *P. citrulli*. ↓, positive regulation; ⊥, negative regulation; T3SS: type III secretion system. Regulatory steps in the model are mainly at the transcriptional level.

In conclusion, we observed that *ClpA* plays a key role in the regulation of many pathogenic factors in *P. citrulli*, such as in vitro motility, in vitro adhesion capacity, antioxidant activity, and T3SS. We also showed that in *P. citrulli*, ClpA does not interact with ClpP, but may has a direct interaction with RepA. *ClpA* negatively regulates *RepA*. RepA is a protein that influences key pathogenicity factors such as pili, T3SS, and virulence proteins in *P. citrulli*, making it a crucial virulence gene (Figure 7). Therefore, Δ*ClpA* affects the expression of virulence factors, such as pili (*pilR*), thus impacting the pathogenicity of *P. citrulli* through its interaction with RepA. Follow-up studies will delve into the *ClpA* regulation by to further regulate these complex regulatory mechanisms.

## Data availability statement

The original contributions presented in the study are included in the article/Supplementary material, further inquiries can be directed to the corresponding author.

## Author contributions

SZ: Data curation, Formal analysis, Writing–original draft. ZY: Conceptualization, Formal analysis, Methodology, Writing–review and editing. WS: Funding acquisition, Methodology, Software, Writing–original draft. CL: Data curation, Project administration, Writing–original draft. SC: Methodology, Software, Writing–original draft. WJ: Software, Writing–review and editing. GW: Validation, Visualization, Writing–review and editing. TY: Data curation, Methodology, Writing–original draft, Writing–review and editing. HB: Writing–review and editing.

## Footnotes

1
https://www.fruitfly.org/seq_tools/promoter.html
