# Supplementary material for: ClpA affects the virulence of Paracidovorax citrulli on melon by regulating RepA
Source: Front Microbiol. 2024 Jul 23;15:1431029. doi: 10.3389/fmicb.2024.1431029 (PMC11300334; doi:10.3389/fmicb.2024.1431029)
Supplement: Supplementary file 1 [file Data_Sheet_1.docx]

Supplementary Material

**Table S1. Primers used in this study.**

| **Primer** | **Sequence(5’-3’)** | **Length** |
| --- | --- | --- |
| **For mutant construction** | | |
| ClpA-F1 | CTCCATCAACTACGCCCTGG | 560 |
| ClpA-R1 | CCACCGTCAGGCGGCCA |  |
| ClpA-F2 | TCCTCGATGTTGGCCGAGC | 180 |
| ClpA-R2 | GAACTCCTTTGTGCTTGCGG |  |
| RepA-F1 | GTGGCAGCTCGGACTGAGAA | 192 |
| RepA-R1 | ATGCGCCAGTCCGGCGCAG |  |
| RepA-R1 | CCACCGTCAGGCGGCCA | 193 |
| RepA-F2 | TCCTCGATGTTGGCCGAGC |  |
| Km-F | TGGCATTCGTTACGCGATC | 1514 |
| Km-R | CCAAGAAGCTCACGTCGTAG |  |
| **For complementary strains construction** | | |
| ClpA-HB-F | CGCTGACCCGCACCC | 2694 |
| ClpA-HB-R | GAACTCCTTTGTGCTTGCGG |  |
| RepA-HB-F | ACCGGACGGAAGCTACCCGC | 1261 |
| RepA-HB-R | TCGATGGCGACATCTCGCTG |  |
| **For qRT-PCR** | | |
| *16s*-F | CCTACGGGAGGCAGCAG | 177 |
| *16s*-R | ATTACCGCGGCTGCTGG |  |
| *pilA*-F | GAACTGATGATCGTGGTGGC | 168 |
| *pilA*-R | CGGAGGACTCGAAGCAGTAG |  |
| *pilR*-F | CGCGCAAGGGTTCCTACA | 98 |
| *pilR*-R | CAGGTCGCCGATCTCGTC |  |
| *fliR*-F | CCATGAGGTGGCCGTTGAT | 173 |
| *fliR*-R | AACTTCGCGTCCTTCTTCG |  |
| *flgM*-F | CCGAAGAGGCCACCAAGACC | 117 |
| *flgM*-R | CATCGAAATCCGCGCTGCTG |  |
| *hrcQ*-F | CGAAGTGGCGGAAACCCTGA | 125 |
| *hrcQ*-R | CGCCCTGGGTCCATTCCAG |  |
| *hrpX*-F | GCGCTCACGCAAATGCT | 175 |
| *hrpX*-R | GGCAAGCTCCTCCTGTCC |  |
| *hrpG*-F | CTGGGCCTGATCCGAAGCTG | 189 |
| *hrpG*-R | CGCGGTTTCCAGCGCATAG |  |
| *trbC*-F | ATGACGCACGTTGATGCTTT | 198 |
| *trbC*-R | GATTCGAGGATGGATTGCAGC |  |
| *virB*-F | CAGCGATCAAGTTGCCTTCG | 144 |
| *virB*-F | CAGCGATCAAGTTGCCTTCG |  |
| qRepA F | GTTTGCAGCTCGGGTTCAAG | 900 |
| qRepA R | GTCATGACGCCTGGCCATAG |  |
| **For bacterial two-hybrid strains construction** | | |
| pTRG-RepA F | AAACCAGAGGCGGCCGGATCCGTGGCA | 900 |
| pTRG-RepA R | GCGCCAGCTCAGACTGAATTCCTACGAC |  |
| pTRG-ssrA F | AAACCAGAGGCGGCCGGATCCATGGCC | 474 |
| pTRG-ssrA R | GCGCCAGCTCAGACTGAATTCTCAGCGG |  |
| pTRG-ClpX F | AAACCAGAGGCGGCCGGATCCTCAGGC | 1266 |
| pTRG-ClpX R | GCGCCAGCTCAGACTGAATTCATGGCCG |  |
| pTRG-ClpP F | AAACCAGAGGCGGCCGGATCCTCAGGG | 609 |
| pTRG-ClpP R | GCGCCAGCTCAGACTGAATTCATGAGC |  |
| pTRG-DnaJ F | AAACCAGAGGCGGCCGGATCCTCAGGT | 1137 |
| pTRG-DnaJ R | GCGCCAGCTCAGACTGAATTCATGTCCA |  |
| pTRG-GrpE F | AAACCAGAGGCGGCCGGATCCTCACTT | 570 |
| pTRG-GrpE R | GCGCCAGCTCAGACTGAATTCATGTCCG |  |
| pBT-ClpA F | TGGCGCGGCCGCATCGAATTCCTCAGT | 2352 |
| pBT-ClpA R | AATTAATTAACTCGAGGATCCATGATTG |  |
| **For GST-pulldown** | | |
| pET30-F-RepA | AAGAAGGAGATATACATATGGCAGCTC | 900 |
| pET30-R-RepA | TCGAGTGCGGCCGCAAGCTTCGACGTG |  |
| pET30-F-ClpX | AAGAAGGAGATATACATATGGCCGAGA | 1266 |
| pET30-R-ClpX | TCGAGTGCGGCCGCAAGCTTGGCCTTCT |  |
| pGEX6p1-ClpA-F | TCCAGGGGCCCCTGGGATCCATGATTGC | 2650 |
| pGEX6p1-ClpA-R | TCGAGTCGACCCGGGAATTCAAGAGGC |  |

#
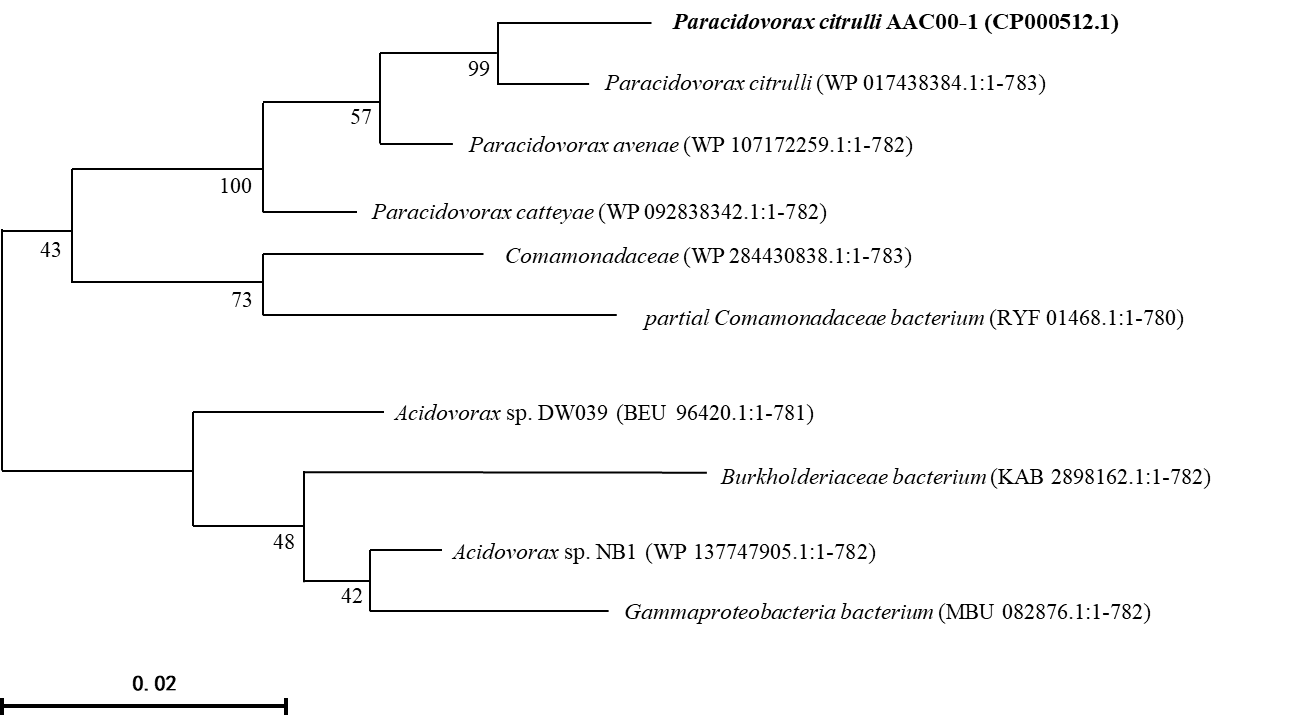
Supplementary Figures

**Figure S1.**Using MEGA X to construct phylogenetic tree, ClpA protein of *Paracidovorax citrulli* AAC00-1 and *Paracidovorax citrulli* (WP017438384.1:1-783) is closely related.


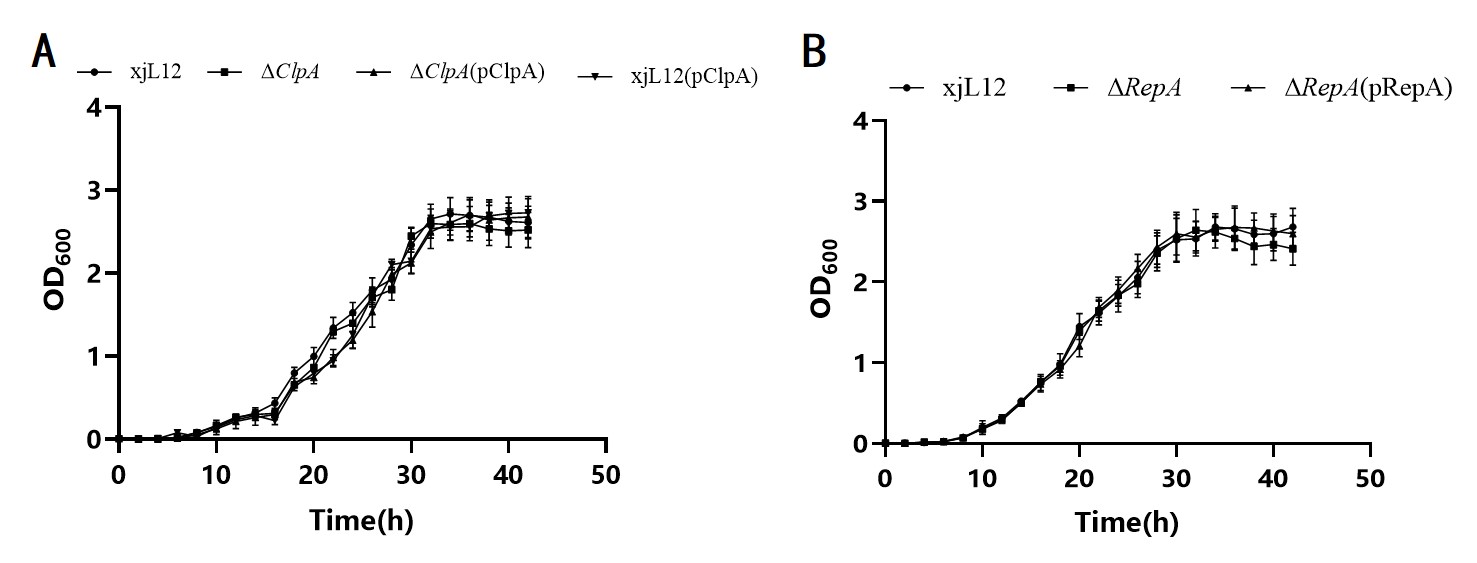


**Figure S2.** The groth curve of *Paracidovorax citrulli* wild-type (WT): xjL12, Δ*ClpA*, Δ*ClpA*(pClpA), Δ*RepA* and Δ*RepA*(pRepA) strains in Luria-Bertani (LB) broth at 28℃. The experiments were performed in triplicate and repeated three times.


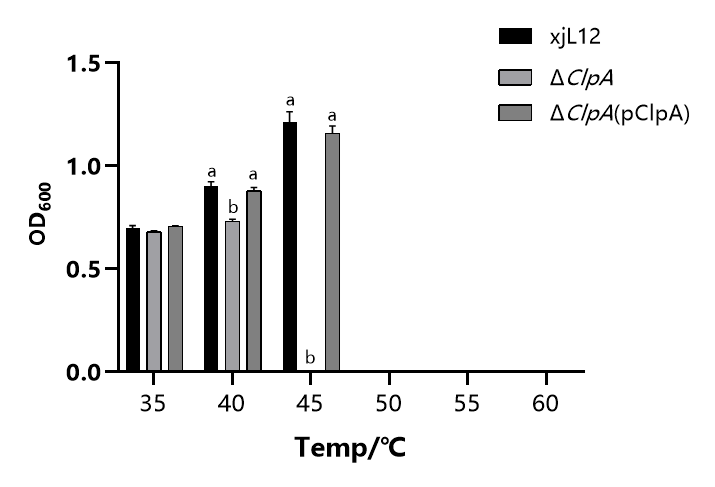


**Fig S3.** The OD_600_ of the survival amount of bacteria under different temperature conditions. *Paracidovorax citrulli* wild-type (WT): xjL12, Δ*ClpA*, Δ*ClpA*(pClpA). After placing each strain in water bath 10 min at different temperatures, 28℃ , 220rpm, shaker and the final OD_600_ value was measured. The experiment was repeated three times.


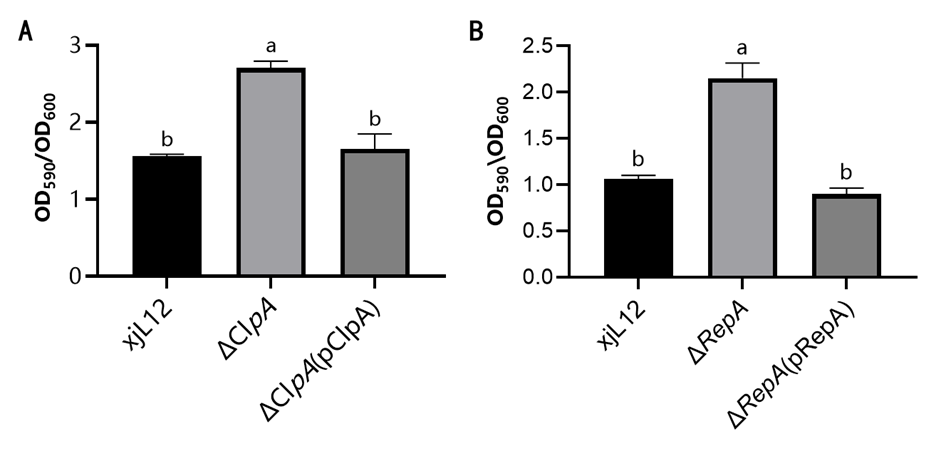


**Fig S4.** (A) Quantitative analysis of biofilm production by *Paracidovorax citrulli* wild-type (WT): xjL12, Δ*ClpA*, Δ*ClpA*(pClpA). (B) Quantitative analysis of biofilm production by *Paracidovorax citrulli* wild-type (WT): xjL12, Δ*RepA*, Δ*RepA*(pRepA) strain. Optical density at 600 nm (OD_600_) was measured for after 48-h liquid cultures of each strain in 12-plate wells and biofilm production were measured after staining with crystal violet using a spectrophotometer at OD_590_/OD_600_. Different lowercase letters indicate a significant difference between treatments. Statistically significant differences were determined by the one-way ANOVA of variance and *p* < 0.05.

**
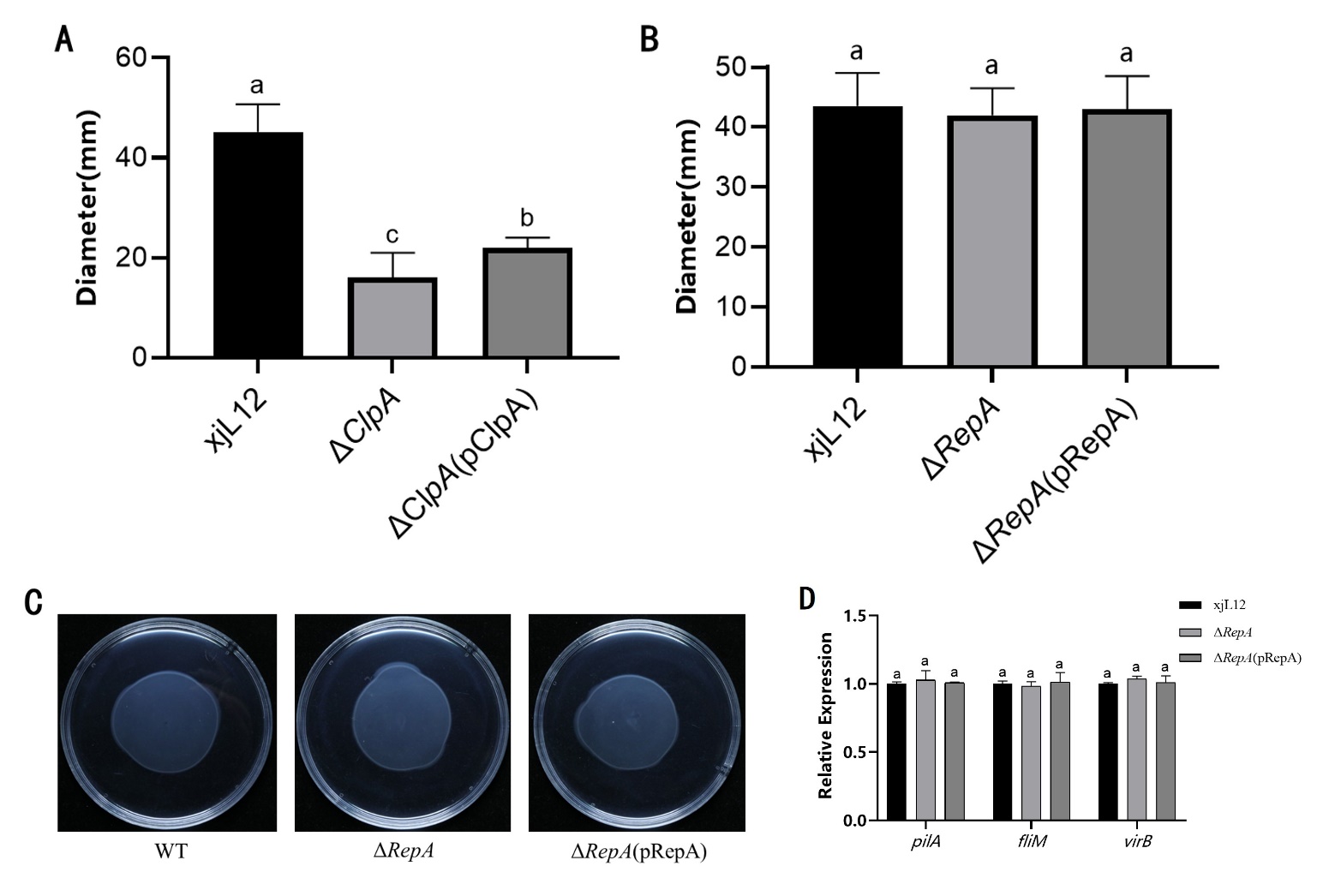
Fig S5.** Quantitative analysis of swimming motility by *Paracidovorax citrulli* wild-type (WT): xjL12~~,~~Δ*RepA*, Δ*RepA*(pRepA) strains. (A) The diameters of WT,Δ*ClpA*, Δ*ClpA*(pClpA) of swimming halos were measured after 48h of incubation. (B) The diameters of WT, Δ*RepA*, Δ*RepA*(pRepA) of swimming halos were measured after 48h of incubation. Different lowercase letters indicate a significant difference between treatments. Statistically significant differences were determined by the one-way ANOVA of variance and *p* < 0.05. (C) Swimming motility of *Paracidovorax citrulli* strains including wild-type (WT): xjL12, Δ*RepA* and Δ*RepA*(pRepA) on 0.3% agar plates at 28°C for 2 days.  (D) Expression level of pathogenicity-related genes *pilA, fliM* and *virB* between *P. citrulli* WT, Δ*RepA* and Δ*RepA*(pRepA) were determined by qRT-PCR. Different lowercase letters indicate a significant difference between treatments. Statistically significant differences were determined by the one-way analysi of variance and *p*< 0.05.
